# Supplementary material for: Prediction of Hardenability Curves for Non-Boron Steels via a Combined Machine Learning Model
Source: Materials (Basel). 2022 Apr 26;15(9):3127. doi: 10.3390/ma15093127 (PMC9103254; doi:10.3390/ma15093127)
Supplement: Supplementary file 1 [file materials-15-03127-s001.zip › materials-1651701-supplementary.pdf]

materials

MDPI

Article

Prediction of Hardenability Curves for Non-Boron Steels via a Combined Machine Learning Model

Xiaoxiao Geng [1], Shuize Wang [1,*], Asad Ullah [2], Guilin Wu [1,3] and Hao Wang [4,*]

[1] Beijing Advanced Innovation Center for Materials Genome Engineering, University of Science and Technology Beijing, Beijing 100083, China; gengxiaoxiao1104@163.com (X.G.); guilinwu@ustb.edu.cn (G.W.)
[2] Department of Mathematical Sciences, Karakoram International University, Gilgit-Baltistan 15100, Pakistan; dr.asadullah@kiu.edu.pk
[3] Yangjiang Branch, Guangdong Laboratory for Materials Science and Technology (Yangjiang Advanced Alloys Laboratory), Yangjiang 529500, China
[4] School of Materials Science and Engineering, University of Science and Technology Beijing, Beijing 100083, China
* Correspondence: wangshuize@ustb.edu.cn (S.W.); xiaohao_2001@163.com (H.W.)

Supplementary Table S1. Range of composition (wt. %), austenitizing temperature (AT, ℃) and Jominy equivalent cooling rate (J_ec, ℃/s) of the steels.

	C	Si	Mn	Cr	Ni	Mo	W	V	Ti	Cu	AT	J_{ec}
Minimum	0.10	0	0	0	0	0	0	0	0	0	800	1.7
Maximum	0.70	1.83	1.89	1.6	1.78	0.54	2.20	0.20	0.09	0.54	930	270

Supplementary Table S2. Number of instances in the training datasets

Datasets	low hardenability steel	high hardenability steel
Number	827	1032

Supplementary Table S3. Optimized parameters and package version of models.

Citation: Geng, X.; Wang, S.; Ullah, A.; Wu, G.; Wang, H. Prediction of Hardenability Curves for Non-boron Steels via a Combined Machine Learning Model. *Materials* **2022**, *15*, 3127. https://doi.org/10.3390/ma15093127

Academic Editors: Szymon Wojciechowski, Antoine Ferreira and Krzysztof Talaśka

Received: 9 March 2022
Accepted: 21 April 2022
Published: 26 April 2022

Publisher's Note: MDPI stays neutral with regard to jurisdictional claims in published maps and institutional affiliations.

Copyright: © 2022 by the authors. Submitted for possible open access publication under the terms and conditions of the Creative Commons Attribution (CC BY) license (https://creativecommons.org/licenses/by/4.0/).

Databases	Optimized Parameters	Package Version
Classification	Random Forest -P 100 -I 100 -num-slots 1 -K 0 -M 1.0 -V 0.001 -S 1	Weka 3.9.5
Low hardenability steels	IBk -K 2 -W 0 -A "weka.core.neighboursearch.LinearNNSearch -A \"weka.core.EuclideanDistance -R first-last\""	Weka 3.9.5
High hardenability steels	Random Forest -P 100 -I 100 -num-slots 1 -K 0 -M 1.0 -V 0.001 -S 1	Weka 3.9.5

Supplementary Table S4. Chemical composition of test set (wt.%)

NO.	Steels	C	Si	Mn	Cr	Ni	Mo	W	V	Ti	Cu
#1	5SiMnMoV	0.57	1	0.95	0	0	0.12	0	0.16	0	0
#2	42SiMn	0.40	1.34	1.21	0.4	0	0	0	0	0	0
#3	40CrNiMoA	0.37	0.3	0.6	0.71	1.43	0.22	0	0	0	0
#4	45CrMnMo	0.455	0.275	0.875	0.975	0	0.2	0	0	0	0
#5	50CrMnVA	0.505	0.275	0.8	0.975	0	0	0	0.075	0	0

Supplementary Table S5. Number of instances in the test set

Test set	#1	#2	#3	#4	#5
Number 6	11	11	11	11	11

Supplementary Note S1. The basic principles of machine learning algorithms

1. Random Forest

Random forests is an ensemble learning method for classification, regression and other tasks that operates by constructing a multitude of decision trees at training time and outputting the class that is the mode of the classes (classification) or mean prediction (regression) of the individual trees [1,2]. Random decision forests correct for decision trees' habit of overfitting to their training set.

Random forests are a way of averaging multiple deep decision trees, trained on different parts of the same training set, with the goal of reducing the variance [3]. This comes at the expense of a small increase in the bias and some loss of interpretability, but generally greatly boosts the performance in the final model.

The training algorithm for random forests applies the general technique of bootstrap aggregating, or bagging, to tree learners. Given a training set X = x1, ..., xn with responses Y = y1, ..., yn, bagging repeatedly (B times) selects a random sample with replacement of the training set and fits trees to these samples[4]:

For b = 1, ..., B:

Sample, with replacement, n training examples from X, Y; call these Xb, Yb.

Train a classification or regression tree fb on Xb, Yb.

After training, predictions for unseen samples x' can be made by averaging the predictions from all the individual regression trees on x':

$$\hat{f} = \frac{1}{B}\sum_{b=1}^{B} f_b(x') \quad \text{(S1)}$$

This bootstrapping procedure leads to better model performance because it decreases the variance of the model, without increasing the bias. This means that while the predictions of a single tree are highly sensitive to noise in its training set, the average of many trees is not, as long as the trees are not correlated. Simply training many trees on a single training set would give strongly correlated trees (or even the same tree many times, if the training algorithm is deterministic); bootstrap sampling is a way of de-correlating the trees by showing them different training sets.

Additionally, an estimate of the uncertainty of the prediction can be made as the standard deviation of the predictions from all the individual regression trees on x':

$$\sigma = \sqrt{\frac{\sum_{b=1}^{B}(f_b(x') - \hat{f})^2}{B-1}} \quad \text{(S2)}$$

The number of samples/trees, B, is a free parameter. Typically, a few hundred to several thousand trees are used, depending on the size and nature of the training set. An optimal number of trees B can be found using cross-validation, or by observing the out-of-bag error: the mean prediction error on each training sample x_i, using only the trees that did not have x_i in their bootstrap sample [5]. The training and test error tend to level off after some number of trees have been fit.

To sum up, Random Forests are an improvement over bagged decision trees. Random forest changes the algorithm for the way that the sub-trees are learned so that the resulting predictions from all of the subtrees have less correlation. It is a simple tweak. In CART, when selecting a split point, the learning algorithm is allowed to look through all variables and all variable values in order to select the most optimal split-point. The random forest algorithm changes this procedure so that the learning algorithm is limited to a random sample of features of which to search.

2. k-Nearest Neighbours

In pattern recognition, the k-nearest neighbors algorithm (k-NN) is a non-parametric method used for classification and regression [6]. k-NN is a type of instance-based learning, or lazy learning, where the function is only approximated locally and all computation is deferred until classification.

Both for classification and regression, a useful technique can be to assign weights to the contributions of the neighbors, so that the nearer neighbors contribute more to the average than the more distant ones. For example, a common weighting scheme consists in giving each neighbor a weight of 1/d, where d is the distance to the neighbor [7]. A commonly used distance metric for continuous variables is euclidean distance, as shown in Formula (S3) [8].

$$\mathrm{d}_{\mathrm{euc}}(x, y) = [\textstyle\sum_{j=1}^{d}(x_j - y_j)^2]^{\frac{1}{2}} = [(x-y)(x-y)^T]^{\frac{1}{2}} \quad \text{(S3)}$$

In *k*-NN regression, the *k*-NN algorithm is used for estimating continuous variables. One such algorithm uses a weighted average of the *k* nearest neighbors, weighted by the inverse of their distance. This algorithm works as follows:

1. Compute the Euclidean distance from the query example to the labeled examples.
2. Order the labeled examples by increasing distance.
3. Find a heuristically optimal number *k* of nearest neighbors, based on Root Mean Square Error. This is done using cross validation.
4. Calculate an inverse distance weighted average with the *k*-nearest multivariate neighbors.

References

1. Ho Tin Kam, Random decision forests, Proceedings of the 3rd International Conference on Document Analysis and Recognition, Montreal, QC, Canada, 1995; 14-16, 278–282. Proceedings of the 3rd International Conference on Document Analysis and Recognition, Montreal, QC, Canada, 1995; 14-16, 278–282.
2. Ho, T.K. The Random Subspace Method for Constructing Decision Forests. IEEE Trans. Pattern Anal. Mach. Intell. 1998, 8, 832–844.
3. Berk R A. Statistical learning from a regression perspective. New York: Springer, 2008.
4. Breiman L. Random forests. Machine Learning, 2001, 45(1): 5-32.
5. James, G.; Witten, D.; Hastie, T.; Tibshirani, R. An Introduction to Statistical Learning; New York: Springer, 2 013; 6, 316-321
6. Altman, N.S. An introduction to kernel and nearest-neighbor nonparametric regression. Am. Statistician 1992, 46, 175–185.
7. Hsu, B. Generalized linear interpolation of language models. In Proceedings of the 2007 IEEE Workshop on Automatic Speech Recognition & Understanding (ASRU), Kyoto, Japan, 9-13 December 2007; pp. 136-140.

8. Vries, A.P.D.; Mamoulis, N.; Nes, N.; Kersten, M. Efficient k-NN search on vertically decomposed Data. In Proceedings of the Proceedings of the 2002 ACM SIGMOD International Conference on Management of Data, Madison, WI, USA, 3-5 June 2002; pp. 322-333.
